# GLP-1 Receptor Agonists and SGLT2 Inhibitors in Stable Kidney Transplantation: Clinical Outcomes from a Cohort of Patients with Post-Transplant Diabetes Mellitus

**DOI:** 10.3390/jcm15010181

**Published:** 2025-12-26

**Authors:** Ricardo E. T. Navarrete, Joana Freitas, Isabel Fonseca, Ana Cunha, Joao Roberto Sa, La Salete Martins

**Affiliations:** 1 Doctoral Program in Medicine, Faculty of Medicine, University of Porto, Alameda Prof. Hernâni Monteiro s/n, 4200-319 Porto, Portugal; dr.ricardonavarrete@gmail.com; 2 Department of Endocrinology and Metabolism, Beneficencia Portuguesa Hospital, Sao Paulo 01323-001, Brazil; 3 Nephrology and Renal Transplant Service, Santo Antonio University Hospital Centre, 4050-342 Porto, Portugal; joanacfreitas@gmail.com (J.F.); isabelfonseca@chporto.min-saude.pt (I.F.); anacristinacunha95@gmail.com (A.C.); 4 Division of Endocrinology, Centro Universitario FMABC, Santo Andre 09060-870, Brazil; jrsa@uol.com.br

**Keywords:** kidney transplantation, diabetes mellitus, SGLT2 inhibitors, GLP-1 receptor agonists, metabolic outcomes, kidney function

## Abstract

**Background:** Despite the lack of formal indication for glucagon-like peptide-1 receptor agonists (GLP-1 RAs) and sodium–glucose cotransporter-2 inhibitors (SGLT2i) in post-transplant diabetes mellitus (PTDM), their use in clinical practice is growing. While robust evidence supporting their use in kidney transplant recipients (KTRs) remains limited, PTDM remains a major driver of adverse outcomes, including cardiovascular morbidity, accelerated graft dysfunction, graft loss, and reduced survival. **Methods:** This retrospective cohort study analyzed adult KTRs with PTDM treated with SGLT2is and/or GLP-1 RAs between 2013 and 2024. Metabolic, kidney, and safety parameters were assessed from baseline to follow-up. **Results:** After a median treatment duration of 1.8 years, glycated hemoglobin (HbA1c) changed from 7.22% to 7.01% (*p* = 0.558), whereas fasting plasma glucose increased from 112.62 mg/dL to 125.01 mg/dL (*p* = 0.03). Body mass index decreased from 27.27 kg/m^2^ to 25.95 kg/m^2^ (*p* < 0.001). The lipid profile improved, with reductions in total cholesterol (*p* < 0.01) and low-density lipoprotein cholesterol (LDL-c, *p* = 0.02). Kidney function remained stable throughout the observation period, and adverse events were infrequent. **Conclusions:** In KTRs with PTDM, GLP-1 RAs and SGLT2is were associated with significant improvements in weight and lipid metabolism, alongside stable kidney function and a favorable safety profile. These findings support the consideration of these agents in the management of PTDM. Prospective studies are warranted to confirm these results.

## 1. Introduction

Post-transplant diabetes mellitus (PTDM) is a well-recognized metabolic complication of kidney transplantation, affecting approximately 15% of recipients and contributing substantially to cardiovascular morbidity, graft loss, and reduced survival [1,2]. It arises from complex disturbances in glucose homeostasis, driven by pancreatic β-cell dysfunction and peripheral insulin resistance, further exacerbated by immunosuppressive regimens [3]. In this context, glycemic management must be tailored to reduced kidney function, a highly pro-atherogenic and pro-inflammatory milieu, and the constraints imposed by immunosuppression, making PTDM a particularly challenging entity to treat [4].

Historically, pharmacological management of PTDM has relied on a limited set of antidiabetic agents, mainly insulin, sulfonylureas, and metformin. Although effective for lowering glucose, these therapies are associated with clinically relevant limitations, including hypoglycemia and weight gain, and they do not provide specific cardiovascular or kidney protection, thereby failing to address the excess cardiovascular and renal risk typical of kidney transplant recipients with diabetes [5]. This therapeutic gap is especially relevant in PTDM, a condition that combines the high baseline risk of chronic kidney disease with the added burden of post-transplant metabolic disturbances.

In parallel, the advent of sodium–glucose cotransporter-2 inhibitors (SGLT2i) and glucagon-like peptide-1 receptor agonists (GLP-1 RAs) has reshaped the management of type 2 diabetes, shifting the paradigm from glucose-centric control toward comprehensive cardiorenal risk reduction [6,7,8]. Large outcome trials have consistently shown that these agents reduce major adverse cardiovascular events, hospitalization for heart failure, and progression of chronic kidney disease, establishing these agents as disease-modifying therapies in high-risk patients with type 2 diabetes [9,10,11]. Through complementary mechanisms—hemodynamic and tubular effects with SGLT2i, and glucose-dependent insulinotropic, glucagon-suppressive and weight-lowering actions with GLP-1 RAs—these drug classes provide benefits that extend well beyond glycemic control [12,13,14,15].

Although SGLT2i and GLP-1 RAs do not yet have specific regulatory approval for PTDM, the substantial overlap between PTDM and type 2 diabetes in terms of metabolic derangements and cardiorenal risk provides a strong biological rationale for cautiously extrapolating the benefits of these agents to carefully selected kidney transplant recipients with PTDM. However, robust, transplant-specific data remain scarce, and important concerns persist regarding potential interactions with immunosuppressive therapies, infection risk, and kidney safety in this vulnerable population [11,12]. Consequently, there is an unmet need for robust real-world evidence to validate the applicability of these agents in the transplant setting and to guide their integration into clinical practice for managing PTDM in KTRs.

To address this gap, we conducted a single-center, retrospective cohort study to evaluate the real-world effectiveness and safety profile of SGLT2i and GLP-1 RAs as add-on therapies in KTRs with PTDM. We assessed their impact on a broad range of cardiometabolic outcomes and kidney function, with the aim of generating clinically relevant data to inform the rational and safe incorporation of these agents into the complex therapeutic landscape of this high-risk population.

## 2. Material and Methods

### 2.1. Study Design and Setting

This retrospective cohort study was conducted at the Adult Kidney Transplant Unit of the Centro Hospitalar Universitário de Santo António in Porto, Portugal, a tertiary university hospital and national reference center with an active transplantation program since 1983. The study included all adult patients who underwent kidney transplantation between August 2013 and April 2024 and subsequently received SGLT2is or GLP-1 RAs for PTDM. Exclusion criteria were multi-organ transplantation, pre-existing diabetes or pre-transplant use of antidiabetic medications, age under 18 years, or incomplete medical records. The final cohort comprised all eligible patients with complete data for analyzing metabolic and kidney outcomes following the initiation of these therapies.

### 2.2. Ethical Considerations

The study protocol was approved by the Institutional Ethics Committee of the Department of Education, Training and Research (DEFI)—Clinical Research Division (Reference No. 2022.264 [209-DEFI/224-CE]) on 19 April 2023. Due to the retrospective, non-interventional nature of the study and the high cardiovascular risk profile of the included patient population, the Ethics Committee granted a waiver of informed consent on 15 June 2023. The final approval for the completed project was issued on 5 September 2024. All data were analyzed in a de-identified format, and the study was conducted in full compliance with institutional data protection policies and the ethical principles of the Declaration of Helsinki.

### 2.3. Data Collection

Clinical data were systematically collected across three domains. Preoperative assessment included demographic characteristics (age at transplantation, sex, self-reported ethnicity, family history of diabetes), anthropometric measures, etiology of end-stage kidney disease, kidney replacement therapy history, donor type, and baseline comorbidities. Postoperative data encompassed immunosuppressive regimens. PTDM-specific parameters included time since diagnosis and detailed antidiabetic treatment history. Laboratory evaluation covered glycemic parameters (fasting plasma glucose, glycated hemoglobin), lipid profile, and kidney function markers (serum creatinine, eGFR). Anthropometric measurements and blood pressure were consistently documented. All parameters were analyzed at baseline (initiation of SGLT2i/GLP-1 RA therapy) and at the most recent follow-up (April 2024) for outcome assessment.

### 2.4. Definitions and Measurements

The diagnosis of PTDM was established based on American Diabetes Association (ADA) criteria, applied no earlier than three months post-transplantation in patients with stable graft function, steady immunosuppression, and no active infection, aligning with international consensus guidelines [16,17]. Diagnosis required meeting at least one of the following criteria: fasting plasma glucose (FPG) ≥ 126 mg/dL on two separate occasions; 2-h plasma glucose ≥ 200 mg/dL during a 75-g oral glucose tolerance test (OGTT); glycated hemoglobin (HbA1c) ≥ 6.5%; or random plasma glucose > 200 mg/dL in the presence of classic hyperglycemic symptoms.

Comorbidities were defined as per established guidelines. Hypertension (HTN) was defined as systolic blood pressure (SBP) ≥ 130 mmHg or diastolic blood pressure (DBP) ≥ 80 mmHg on repeated measurements. Dyslipidemia (DL) management adhered to Kidney Disease: Improving Global Outcomes (KDIGO) recommendations, targeting a low-density lipoprotein cholesterol (LDL-C) level of <55 mg/dL or a ≥50% reduction from baseline for patients classified at very high cardiovascular risk [18]. Kidney function was assessed using the 2021 Chronic Kidney Disease Epidemiology Collaboration (CKD-EPI) equation, consistent with the National Kidney Foundation–Kidney Disease Outcomes Quality Initiative (NKF-K/DOQI) guidelines [19].

All laboratory parameters were retrieved retrospectively from the institutional transplant database. All laboratory measurements were performed in the institution’s central laboratory and reported using conventional clinical units. Glycemic parameters included fasting plasma glucose (mg/dL) and glycated hemoglobin (HbA1c, %), measured by high-performance liquid chromatography (HPLC) and standardized to DCCT/NGSP criteria. The lipid profile—total cholesterol, low-density lipoprotein cholesterol (LDL-C), high-density lipoprotein cholesterol (HDL-C), and triglycerides—was expressed in mg/dL. Kidney function was assessed using serum creatinine (mg/dL) and estimated glomerular filtration rate (eGFR, mL/min/1.73 m^2^). Anthropometric and hemodynamic measurements included body weight (kg), body mass index (kg/m^2^), and blood pressure (mmHg). All assays were performed using automated analyzers routinely employed in our clinical laboratory.

### 2.5. Study Outcomes

This study evaluated the metabolic, kidney, and safety outcomes following the initiation of SGLT2i and/or GLP-1 RA in KTRs with PTDM. Primary outcomes assessed metabolic efficacy through changes in fasting plasma glucose (FPG, mg/dL), glycated hemoglobin (HbA1c, %), lipid profile, body weight (kg), and body mass index (BMI, kg/m^2^), while kidney outcomes included changes in estimated glomerular filtration rate (eGFR, mL/min/1.73 m^2^), serum creatinine (mg/dL), and serum urea (mg/dL). Serum uric acid was evaluated as an additional metabolic parameter. Secondary outcomes focused on safety and tolerability, documenting the incidence and severity of adverse events from medical records, including urinary and genital infections, gastrointestinal symptoms, hypoglycemia, and other treatment-related complications.

### 2.6. Statistical Analysis

Descriptive statistics characterized baseline and outcome variables. Continuous, normally distributed data were expressed as mean ± standard deviation (SD), and non-normal data as median with interquartile range (IQR). The Shapiro–Wilk test assessed normality. Categorical variables were presented as frequencies and percentages. Within-group comparisons (before vs. after treatment) for continuous variables employed paired *t*-tests for normal data or Wilcoxon signed-rank tests for non-parametric data. Between-group comparisons for categorical variables used chi-square or Fisher’s exact tests, as appropriate. For the prespecified subgroup analysis, differences across the three treatment groups (GLP-1 RA add-on, SGLT2i add-on, and combination add-on) were analyzed using the Friedman test. All analyses were performed using SAS software version 9.4 (SAS Institute Inc., Cary, NC, USA, 2013), with a significance level set at *p* < 0.05 (two-tailed).

## 3. Results

### 3.1. Cohort Selection and Composition

**Figure 1:**
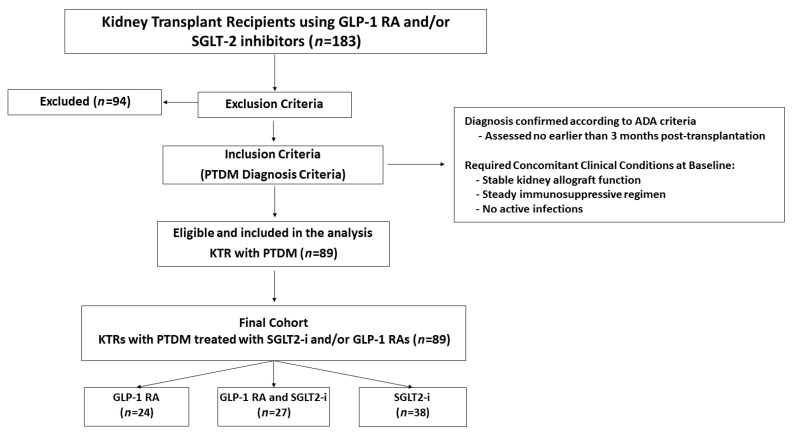
Flowchart of Cohort Selection and Final Sample Composition. Abbreviations: ADA: American Diabetes Association; GLP-1 RA: Glucagon-like Peptide-1 Receptor Agonist; KTR: Kidney Transplant Recipient; PTDM: Post-Transplant Diabetes Mellitus; SGLT2-i: Sodium-Glucose Cotransporter-2 Inhibitor.

From an initial screening of 183 KTRs treated with GLP-1 RAs and/or SGLT2is, 94 patients were excluded based on predefined criteria. The main reasons for exclusion were pre-existing type 2 diabetes (T2DM; *n* = 52), absence of a confirmed diabetes diagnosis (*n* = 19), and incomplete medical records (*n* = 10). Other exclusions comprised type 1 diabetes (T1DM; *n* = 5), impaired fasting glucose (IFG; *n* = 3), transient hyperglycemia (*n* = 2), age under 18 at transplantation (*n* = 1), history of multiple organ transplantation (*n* = 1), and one case of MODY8/CEL-MODY [Maturity-Onset Diabetes of the Young type 8 caused by mutations in the carboxyl ester lipase (CEL) gene]. The final cohort consisted of 89 KTRs with confirmed PTDM who met all inclusion criteria. All patients were on a background antidiabetic regimen at the time of initiation of the study drugs. For the purpose of subgroup analysis, the cohort was stratified based on the type of advanced therapy introduced: those who had a GLP-1 RA added to their regimen (*n* = 24), those who had an SGLT2i added (*n* = 38), and those who had both drug classes initiated (*n* = 27), as detailed in Figure 1.

### 3.2. Baseline Characteristics of the Cohort

The final study cohort consisted of 89 KTRs with PTDM, with a median age of 60.0 years (IQR: 55.0–67.0), and a predominance of male patients (64%). The burden of baseline comorbidities was substantial: hypertension was present in 98% of patients, dyslipidemia in 98%, and heart failure in 26%. Additionally, 54% reported a family history of diabetes. A notable discrepancy emerged in the assessment of obesity. While medical records documented obesity in 56% of patients, objective BMI evaluation classified 28% as obese (BMI ≥ 30 kg/m^2^) and 43% as overweight (BMI 25–29.9 kg/m^2^), indicating that 71% of the cohort had excess weight. This inconsistency likely reflects the subjective nature of routine clinical documentation, where obesity may have been classified based on physical appearance or perceived cardiometabolic risk rather than standardized anthropometric criteria. The cohort had a median baseline BMI of 27.25 kg/m^2^ (IQR: 23.92–30.76), consistent with an overall overweight profile.

Regarding pre-transplant history, most patients (76%) underwent hemodialysis as kidney replacement therapy, while 16% received peritoneal dialysis, and 8% underwent preemptive transplantation.

The median duration of kidney replacement therapy was 3.92 years (IQR: 1.00–6.29 years). The primary etiologies of end-stage kidney disease were glomerular diseases (27%), polycystic kidney disease (20%), tubulointerstitial diseases (10%), other identified causes (12%), and unknown causes (31%). Most recipients (78%) received kidneys from deceased donors. At study inclusion, the median time since transplantation was 9.1 years (IQR: 4.3–13.8 years), and the median duration of PTDM was 8.17 years (IQR: 4.42–14.25 years).

Immunosuppressive regimens followed standard protocols: corticosteroids were used in 94% of patients, tacrolimus in 84%, mycophenolate mofetil in 87%, and cyclosporine in 15%. Prior to initiating SGLT2is and/or GLP-1 RAs—the central interventions under investigation—patients were managed with various antidiabetic agents, including biguanides (33%), sulfonylureas (11%), DPP-4 inhibitors (39%), and insulin (48%). Comprehensive baseline characteristics are detailed in Table 1, organized by clinical features, pre-transplant history, post-transplantation profile, and PTDM management.

### 3.3. Evolution of Antidiabetic Treatment Patterns

**Figure 2:**
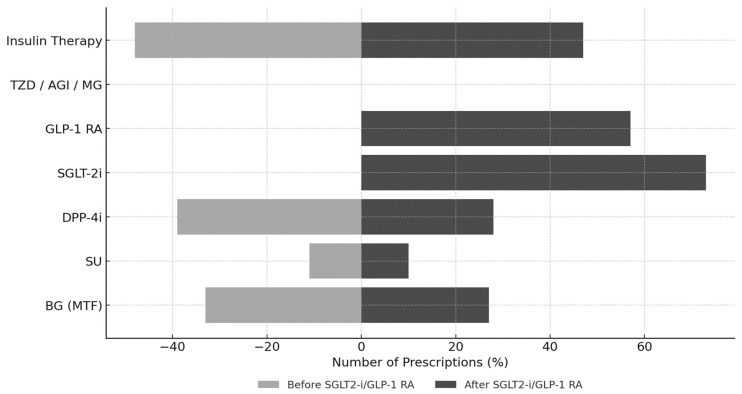
Distribution of antidiabetic drug classes before and after the initiation of SGLT2i and/or GLP-1 RA in KTRs with PTDM (*n* = 89). Abbreviations: AGI: Alpha-Glucosidase Inhibitors; BG: Biguanides; DPP-4i: Dipeptidyl Peptidase-4 Inhibitors; GLP-1 RA: Glucagon-Like Peptide-1 Receptor Agonists; MG: Meglitinides; SGLT-2i: Sodium-Glucose Cotransporter-2 Inhibitors; SU: Sulfonylureas; TZD: Thiazolidinediones. Note: TZD, AGI and MG: Not Reported.

Figure 2 illustrates the evolution of antidiabetic therapy following the initiation of SGLT2is and/or GLP-1 RAs. After a median treatment duration of 1.8 years (IQR: 0.9–3.5 years), a notable shift in prescribing patterns emerged. SGLT2is were prescribed to 65 patients (73%) and GLP-1 RAs to 51 patients (57%), with 27 patients (30% of the cohort) receiving combination therapy with both classes. Regarding specific agents, the GLP-1 RAs used were dulaglutide (*n* = 25), semaglutide (*n* = 15), liraglutide (*n* = 10), and exenatide (*n* = 1). Among SGLT2is, the prescribed agents were dapagliflozin (*n* = 49), empagliflozin (*n* = 12), and canagliflozin (*n* = 4).

This shift toward newer agents was accompanied by a decline in conventional therapies: metformin use decreased from 33% to 27% and DPP-4 inhibitors from 39% to 28%, while the use of sulfonylureas (11% to 10%) and insulin (48% to 47%) remained largely stable.

Across the three therapeutic cohorts, a broadly similar pattern of background treatment was observed, with metformin and insulin generally maintained while less preferred agents—most notably DPP-4 inhibitors—were progressively de-escalated. Nonetheless, distinct prescribing profiles emerged within each group. In the GLP-1 RA cohort (*n* = 24), sulfonylureas were frequently withdrawn, whereas insulin was typically continued, suggesting a strategy of intensification via incretin-based therapy rather than further escalation of secretagogues. In the SGLT2i cohort (*n* = 38), insulin and DPP-4 inhibitors were more commonly maintained, with SGLT2i introduced as an add-on to existing regimens; this pattern is consistent with the substantial burden of heart failure in this subgroup (23/38, 60%), reflecting a therapeutic approach aimed at simultaneously optimizing glycemic control and cardiovascular risk. In the combination therapy cohort (GLP-1 RA + SGLT2i, *n* = 27), metformin and insulin were broadly preserved, sulfonylureas were discontinued in all prior users, and DPP-4 inhibitors were occasionally retained on a transient basis, most plausibly during the overlap period required for GLP-1 RA dose titration and SGLT2i initiation. Taken together, these prescribing patterns reflect a progressive shift from traditional antidiabetic agents toward contemporary therapies with established cardiorenal protective properties in the management of PTDM.

### 3.4. Metabolic and Kidney Outcomes

To assess the clinical impact of the observed therapeutic shift, we analyzed key metabolic and kidney parameters. Following the initiation of SGLT2i and/or GLP-1 RA therapy, several metabolic parameters evolved in a favorable direction. HbA1c decreased from 7.22% to 7.01% (Δ = −0.21%, *p* = 0.558), while FPG from 112.62 ± 26.03 to 125.01 ± 30.60 mg/dL (*p* = 0.03). Beyond glycemic parameters, lipid profiles improved notably against a background of prevalent statin therapy (atorvastatin [*n* = 51], rosuvastatin [*n* = 22], simvastatin [*n* = 16]), with ezetimibe supplementation in 42% of patients. Total cholesterol decreased from 175.53 to 162.46 mg/dL (*p* < 0.01) and LDL cholesterol from 87.23 to 80.92 mg/dL (*p* = 0.02). Triglyceride levels moved from 187.67 to 179.72 mg/dL (*p* = 0.45), and HDL cholesterol increased from 48.69 ± 13.42 to 50.33 ± 12.84 mg/dL (*p* = 0.07). All patients continued their existing statin regimens throughout follow-up, with no modifications in type, dose, or intensity, indicating consistent lipid-lowering management during the study period. Concurrently, anthropometric measures showed substantial benefits. Mean body weight decreased by 3.47 kg (*p* = 0.03), accompanied by a reduction in BMI from 27.27 to 25.95 kg/m^2^ (*p* < 0.001). The proportion of patients with obesity (BMI ≥ 30) decreased from 28% to 17%, while those with normal weight (BMI < 25) increased from 32% to 44% (*p* = 0.106 for categorical shift). This was accompanied by improved blood pressure control, with a significant reduction in systolic blood pressure (*p* < 0.01) and a trend toward lower diastolic pressure (*p* = 0.08). Blood pressure control was achieved solely through dose optimization of the existing antihypertensive regimen, without introducing new drug classes or agents. Regarding kidney safety, function remained stable over a median treatment period of 1.8 years. Serum creatinine showed minimal change (−0.02 mg/dL), while mean eGFR increased from 52.74 ± 20.61 to 54.81 ± 21.37 mL/min/1.73 m^2^ (*p* = 0.11), equivalent to an annualized change of +0.83 mL/min/1.73 m^2^/year. This functional stability was paralleled by the absence of any meaningful progression in proteinuria, whether assessed as albuminuria or total protein excretion. Both urine albumin-to-creatinine ratio (UACR) and urine protein-to-creatinine ratio (UPCR) exhibited non-normal distributions and are therefore presented as median (IQR). Baseline UACR and UPCR were 0.79 (0.15–7.43) mg/g and 3.43 (1.29–10.82) mg/g, respectively, compared with 0.71 (0.28–13.21) mg/g and 3.23 (1.59–10.58) mg/g at follow-up. Neither parameter changed significantly over time (UACR: *p* = 0.91; UPCR: *p* = 0.48; Wilcoxon signed-rank test). A comprehensive summary of these outcomes is provided in Table 2. This overall metabolic and kidney safety profile provides important context for the adverse event analysis that follows.

### 3.5. Subgroup Analyses by Type of Antidiabetic Therapy Initiated

Subgroup analyses compared outcomes across three treatment strategies: GLP-1 RA monotherapy, SGLT2i monotherapy, and combination therapy. Overall, each therapeutic approach demonstrated a consistent trend towards metabolic improvement while preserving kidney function.

Regarding glycemic control, HbA1c levels decreased across all treatment arms, though these reductions did not reach statistical significance. The GLP-1 RA group showed a decline from 7.42% to 7.03% (*p* = 0.681), the SGLT2i group from 7.31% to 6.89% (*p* = 0.098), and the combination therapy group from 7.22% to 7.01% (*p* = 0.558). A more pronounced and uniform effect was observed in anthropometric parameters. Weight-related measures improved significantly in all subgroups. BMI decreased from 27.86 to 25.15 kg/m^2^ in the GLP-1 RA group (*p* = 0.05), from 26.30 to 25.35 kg/m^2^ in the SGLT2i group (*p* = 0.03), and from 27.27 to 25.95 kg/m^2^ with combination therapy (*p* < 0.001).

This anthropometric benefit was complemented by improvements in hemodynamic profile. Systolic blood pressure (SBP) reductions were observed in all groups, reaching statistical significance in the SGLT2i monotherapy group (from 143.7 mmHg to 136.9 mmHg, *p* = 0.0085), with strong trends in the GLP-1 RA group (from 142.9 mmHg to 134.7 mmHg, *p* = 0.065) and combination therapy group (from 145.9 mmHg to 138.6 mmHg, *p* = 0.123). Diastolic blood pressure (DBP) also showed favorable trends, with a significant reduction in the combination therapy group (from 81.6 mmHg to 73.0 mmHg, *p* = 0.040), a trend toward significance in the SGLT2i group (from 76.0 mmHg to 73.3 mmHg, *p* = 0.054), and a non-significant reduction in the GLP-1 RA group (from 74.8 mmHg to 70.4 mmHg, *p* = 0.335).

**Figure 3:**
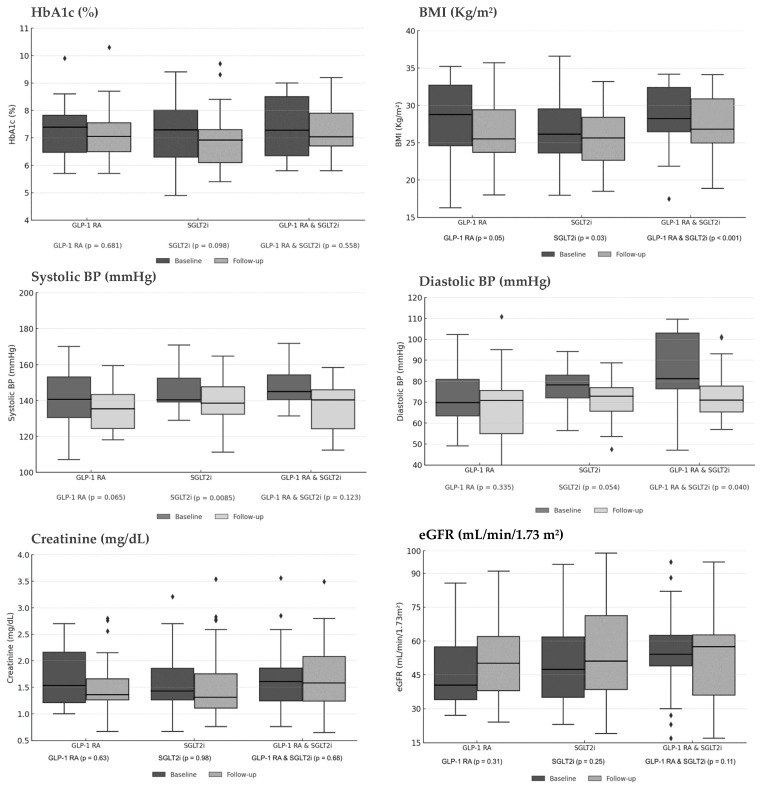
Boxplots illustrating the changes in HbA1c, BMI, Creatinine, eGFR, and Systolic/Diastolic Blood Pressure from baseline to follow-up across treatment subgroups. Abbreviations: BMI: Body Mass Index; BP: Blood pressure; eGFR: estimated Glomerular Filtration Rate; HbA1c: Hemoglobin A1c; GLP-1 RA: Glucagon-Like Peptide-1 Receptor Agonist; SGLT2i: Sodium-Glucose Cotransporter-2 Inhibitor.

Crucially, all therapeutic strategies demonstrated a consistent profile of kidney safety. Kidney function remained stable throughout the study period. Serum creatinine levels showed minimal variation: from 1.51 to 1.43 mg/dL (*p* = 0.63) in the GLP-1 RA group, from 1.45 to 1.36 mg/dL (*p* = 0.98) in the SGLT2i group, and from 1.59 to 1.57 mg/dL (*p* = 0.68) with combination therapy. Similarly, eGFR demonstrated modest improvements: from 42.24 to 50.04 mL/min/1.73 m^2^ (*p* = 0.31), from 47.62 to 50.86 (*p* = 0.25), and from 52.74 to 54.81 (*p* = 0.11) in the respective treatment groups. Figure 3 illustrates the longitudinal changes in glycemic, anthropometric, and kidney parameters across treatment subgroups, comparing baseline with final follow-up values.

### 3.6. Adverse Events According to Treatment Exposure

Among the 89 patients evaluated, the overall safety profile of SGLT2i and GLP-1 RA therapy was favorable. Adverse events were infrequent and predominantly mild to moderate in severity. Regarding infectious complications, no cases of genital infections were reported. Urinary tract infections (UTIs) were documented in 14 individuals (15.7%), with a higher incidence in women (*n* = 10) than men (*n* = 4). The clinical course was benign in the vast majority of cases; most were asymptomatic (*n* = 8) or mild (*n* = 5), with a single moderate case (1.1%) leading to treatment discontinuation. Critically, no episodes of urosepsis occurred. Gastrointestinal tolerability was generally good. No cases of diarrhea or altered bowel habits were reported. Nausea was reported in five patients (5.6%), characterized as mild to moderate, responsive to antiemetic therapy, and self-limited to injection days. Nonetheless, this led to dose reduction and subsequent discontinuation in four patients—two on dulaglutide and two on semaglutide—to avoid compromising adherence in the context of post-transplant polypharmacy. The risk of hypoglycemia was low. Events occurred in six patients (6.7%), distributed across all treatment groups. Half of these events were asymptomatic. It is noteworthy that five of the six affected patients were concurrently using insulin; despite this, no treatment discontinuations resulted from hypoglycemia. Finally, no cases of injection site reactions, volume depletion, or acute kidney injury were observed during the study period, reinforcing the overall safety of these therapies in this vulnerable population.

## 4. Discussion

This real-world cohort study demonstrates that the use of SGLT2is and/or GLP-1 RAs in KTRs with PTDM, although not yet specifically approved for this indication, is associated with a multifaceted cardiometabolic benefit and a manageable safety profile over a median treatment exposure of 1.8 years. We observed significant improvements in weight, lipid metabolism, and blood pressure, alongside a trend toward improved glycemic control and, notably, stable kidney function. These findings persisted in subgroup analyses and occurred with a low frequency of adverse events. Collectively, these results provide clinical validation for the theoretical pathophysiological rationale underpinning the use of these agents in PTDM.

This rationale stems from the complementary mechanisms of SGLT2is and GLP-1 RAs that directly address the core pathophysiology of PTDM. SGLT2is and GLP-1 RAs have transformed the management of T2DM globally, providing not only effective glycemic control but also offering crucial cardiorenal protection. More recently, their use has expanded to diabetes associated with chronic kidney disease, with emphasis placed on preserving kidney function. Although not yet formally approved for PTDM, these agents are increasingly used in real-world clinical practice. From a mechanistic standpoint, SGLT2is and GLP-1 RAs exhibit complementary actions highly relevant to PTDM. SGLT2is and GLP-1 RAs exhibit complementary actions that are highly relevant to the core pathophysiology of PTDM, extending far beyond simple glycemic control. SGLT2is, by promoting glycosuria, not only reduce glucotoxicity—alleviating endoplasmic reticulum stress and β-cell apoptosis—but also induce a state of negative energy balance that reduces the release of free fatty acids and inflammatory adipokines from visceral fat, directly improving corticosteroid-induced peripheral insulin resistance. Furthermore, by activating tubuloglomerular feedback, they reduce intraglomerular hyperfiltration, protecting the graft. Conversely, GLP-1 RAs circumvent β-cell dysfunction by providing a glucose-dependent insulinotropic stimulus, which is crucial in countering the calcineurin-mediated inhibition of insulin secretion by tacrolimus.

Their suppression of glucagon counteracts steroid-exacerbated hepatic gluconeogenesis, while their central and gastrointestinal actions promote satiety and weight loss, positively modulating insulin sensitivity. Together, these classes not only address the core metabolic defects of PTDM but also offer pleiotropic cardiorenal protection, impacting outcomes that go far beyond glycemia. Notably, in a cohort of kidney transplant recipients, Kukla et al. reported that initiation of liraglutide did not lead to relevant changes in immunosuppressive drug dosing, supporting a favorable interaction profile of GLP-1 RAs in this setting [20].

In our cohort, the prescription of GLP-1 RAs and SGLT2is reflects a growing preference for agents with established cardiorenal benefits in other populations with diabetes, over older antidiabetic drugs, which may lack protective effects and pose risks of significant side effects—especially in this clinically complex and historically underrepresented population. Our results suggest that SGLT2is and GLP-1 RAs may be associated with a trend toward improved metabolic control and preservation of kidney function in PTDM. Although a transient increase in fasting plasma glucose was observed, a slight reduction in mean HbA1c (−0.21%) was documented, indicating mild improvement in overall glycemic control. These findings align with glycemic and kidney benefits reported in large-scale trials such as EMPA-KIDNEY Outcome Study [7] and DAPA-CKD Study [9], where SGLT2is contributed to improved glucose regulation and delayed kidney disease progression in high-risk groups. Although our cohort is specific and clinically complex, the observed improvements may be further validated in larger, more diverse populations and with longer treatment exposure.

Significant reductions in total cholesterol (−13.07 mg/dL, *p* < 0.01) and LDL-c (−6.31 mg/dL, *p* = 0.02) were observed, along with a marginal increase in HDL-c (+1.64 mg/dL, *p* = 0.07), suggesting additional metabolic benefits. However, these findings should be interpreted with caution, as they occurred without changes in statin potency, switching of lipid-lowering agents, or dose intensification, and other relevant variables—such as structured physical activity plans and potential dietary modifications—were not systematically recorded. The modest rise in HDL-c is consistent with findings from DECLARE–TIMI 58 [8] and CANVAS [10] trials, although SGLT2i therapy in those studies was associated with slight increases in LDL-c and total cholesterol. Nevertheless, overall cardiovascular benefits were maintained. The lipid improvements in our cohort may be partly attributable to GLP-1 RA therapy, either as monotherapy or in combination with SGLT2is, consistent with results from the LEADER, SUSTAIN-6, and REWIND trials, where lipid changes, though generally modest, contributed to cardioprotective effects alongside improved glycemic control, weight reduction, and anti-inflammatory properties [11,13,15].

Our cohort consisted of patients with PTDM who, at baseline, had stable graft function, were on a steady immunosuppressive regimen, and had no active infections. Throughout the observation period, kidney function remained stable in this population, with a modest increase in eGFR (+2.07 mL/min/1.73 m^2^) and slight reductions in serum urea (−3.75 mg/dL) and creatinine (−0.02 mg/dL). These findings are consistent with nephroprotective trends observed in EMPA-KIDNEY and DAPA-CKD, where SGLT2is slowed chronic kidney disease progression in patients with and without T2DM. The reduction in serum uric acid (−0.34 mg/dL, *p* = 0.03) aligns with findings from Halden et al. [21], suggesting ancillary metabolic benefits beyond glucose control. Adding to the preservation of graft function, the renoprotective profile associated with these contemporary glucose-lowering agents was also reflected in the behavior of proteinuria markers. Median uACR values moved from 0.79 to 0.71 mg/g (*p* = 0.91), and median uPCR values from 3.43 to 3.23 mg/g (*p* = 0.48), remaining within the normal physiological range over the entire observation period. Rather than the progressive increase in proteinuria typically anticipated in PTDM, this stable pattern aligns with the renoprotective trajectory reported in the CREDENCE trial [9], even though our cohort started with much lower baseline proteinuria (median uACR 0.79 mg/g versus approximately 900 mg/g in CREDENCE). This pattern is in line with observations from other transplant cohorts: Fructuoso et al. [22] documented a median ΔuACR of −16 mg/g at six months, while in the short-term series by Schwaiger et al. [23], proteinuria remained stable, with no significant change after four weeks of therapy.

Anthropometric outcomes revealed a discrepancy between subjective classifications and objective measures: although medical records indicated obesity in 56% of patients, baseline data showed the cohort was predominantly overweight, with a mean BMI of 27.27 ± 4.86 kg/m^2^ and mean body weight of 73.95 ± 14.89 kg. By the end of the study, a mean weight loss of 3.47 kg (*p* = 0.03) and BMI reduction of 1.32 kg/m^2^ (*p* < 0.001) were observed, consistent with results from Kim et al. [24] and Vigara et al. [25] in KTRs treated with GLP-1 RAs or SGLT2is. These anthropometric improvements should, however, be interpreted with caution, as GLP-1 RA dosing followed routine clinical practice rather than a standardized protocol, and concomitant lifestyle factors, including dietary changes and physical activity, were not systematically assessed.

Reductions in systolic (−6.03 mmHg; *p* < 0.01) and diastolic (−2.06 mmHg; *p* = 0.08) blood pressure further underscore the hemodynamic benefits of these agents, in line with findings from DECLARE–TIMI 58 and CANVAS trials [8,10].

Large-scale cardiovascular outcome trials with GLP-1 RAs—such as LEADER (liraglutide), SUSTAIN-6 (semaglutide), and REWIND (dulaglutide)—have demonstrated significant HbA1c reductions and robust cardiorenal protection in high-risk T2DM populations [10,11,12,13,14,15]. LEADER reported a 13% relative risk reduction in major adverse cardiovascular events (MACE), while SUSTAIN-6 showed delayed progression of diabetic nephropathy. These benefits are echoed in transplant-focused studies such as Sato et al. [26], which reported improved glycemic and kidney outcomes with GLP-1 RA use, and Mallik et al., which corroborated reductions in weight and insulin requirements among transplant recipients [27].

Safety outcomes in our cohort were favorable. Urinary tract infections occurred in 14 patients (16%), predominantly mild or moderate, consistent with rates reported by Lemke et al. [28] and Song et al. [29] in KTRs using SGLT2is. No genital infections were recorded, contrasting with the 3.3% incidence reported by Halden et al. [21]. Treatment discontinuation due to intolerance occurred in 7 patients (8%), primarily with GLP-1 RAs (dulaglutide and semaglutide), aligning with discontinuation rates described by Mallik et al. [27]. Hypoglycemia was reported in 6 patients (7%), all using concomitant insulin, consistent with observations by Pinelli et al. [30] and Liou et al. [31] regarding increased hypoglycemia risk with insulin combined with SGLT2is or GLP-1 RAs in transplant recipients. Importantly, no cases of acute kidney injury or volume depletion were observed, contrasting with findings by Schweiger et al. [23], who reported AKI episodes among SGLT2i users.

### Strengths and Limitations

This study provides real-world evidence on the safety and clinical effectiveness of incorporating newer antidiabetic agents into the management of post-transplant diabetes. These findings should, however, be interpreted in light of the study’s observational nature. As a retrospective analysis, this study is inherently subject to methodological constraints, including the absence of randomization and blinding, as well as a limited ability to explore detailed dose–response relationships. In addition, bone-specific assessments were not systematically collected in the subgroup treated with GLP-1 RAs; therefore, we could not evaluate bone health or musculoskeletal safety, which is particularly relevant in the context of emerging concerns that long-term GLP-1 RA therapy may be associated with sarcopenia and potential bone loss. This gap underscores the need for dedicated prospective studies addressing musculoskeletal outcomes in this population.

Moreover, our results pertain to clinically stable KTRs with established PTDM who survived beyond the early post-transplant period and thus may not reflect outcomes in higher-risk patients who died or experienced graft deterioration before treatment initiation. Future prospective studies with systematic longitudinal follow-up will be essential to clarify temporal and mechanistic relationships. Finally, because this analysis focused primarily on graft function, kidney stability, and metabolic control, macrovascular complications were not captured and warrant dedicated evaluation in subsequent research.

Notwithstanding these limitations, this study offers several compelling strengths and is among the first to evaluate a well-characterized cohort of KTRs with PTDM and assess the real-world use of SGLT2i and GLP-1 RA over a meaningful longitudinal period. Our analysis leverages a large, well-characterized cohort from a high-volume tertiary transplant center, with data systematically captured over a substantial observational window of nearly 11 years. The use of a structured institutional registry comprising consecutive, unselected patients enhances the representativeness of our findings and their generalizability to real-world clinical practice.

A pivotal strength is our specific focus on the understudied PTDM population. By delineating this clinically distinct group from KTRs with pre-existing T2DM, we deliver targeted insights into the glycemic, metabolic, and kidney outcomes for these high-risk individuals, thereby filling a critical evidence gap. Furthermore, with a median treatment exposure of 1.8 years, this investigation moves beyond short-term effects, offering valuable data on the intermediate-term clinical utilization and outcomes associated with these modern therapies in a transplant setting. Collectively, these elements—the long-term, real-world data, the dedicated focus on PTDM, and the robust cohort design—provide a substantial and unique contribution to the field, directly informing the evolving management strategies for this vulnerable patient population.

## 5. Conclusions

In KTRs with PTDM, SGLT2is and GLP-1 RAs were associated with a favorable cardiometabolic profile and stable kidney function, as reflected by unchanged eGFR, serum creatinine, and proteinuria markers (uACR and uPCR), demonstrating a safety profile consistent with their use in the general population. These real-world findings support the integration of these agents into the therapeutic arsenal for PTDM. Prospective studies are now warranted to confirm their efficacy on long-term cardiovascular and kidney outcomes in this unique high-risk population.

## Figures and Tables

**Table 1:** Baseline demographic and clinical characteristics of the study cohort.

Variable	Measure	Value
**Clinical features**		
Age, years	Median [IQR]	60 [55.0–67.0]
	Mean ± SD (range)	59.95 ± 10.92 (25–81)
Gender, male/female	*n* (%)	57 (64%)/32 (36%)
Family history of DM	*n* (%)	48 (54%)
BMI, kg/m^2^	Median [IQR]	27.25 [23.92–30.76]
	Mean ± SD (range)	27.27 ± 4.86 (16.26–36.57)
Comorbidities pre-SGLT2-i/GLP-1 RA initiation		
Hypertension	*n* (%)	87 (98%)
Dyslipidemia	*n* (%)	87 (98%)
Obesity (as recorded in medical charts)	*n* (%)	50 (56%)
Obesity (based on BMI ≥ 30 kg/m^2^)	*n* (%)	23 (26%)
Heart Failure	*n* (%)	23 (26%)
Time since KT until study inclusion, years	Median [IQR]	9.1 [4.3–13.8]
	Mean ± SD (range)	9.67 ± 6.28 (0.25–29.9)
**Pre- and Post-Transplantation Clinical Profile**		
Etiology of ESKD		
Glomerular diseases	*n* (%)	24 (27%)
Polycystic disease	*n* (%)	18 (20%)
Tubulointerstitial disease	*n* (%)	9 (10%)
Other causes	*n* (%)	11 (12%)
Unknown	*n* (%)	27 (31%)
Dialysis modality *		
Hemodialysis	*n* (%)	68 (76%)
Peritoneal Dialysis	*n* (%)	14 (16%)
Not on dialysis	*n* (%)	7 (8%)
Time on KRT, years	Median [IQR]	3.92 [1.0–6.29]
	Mean ± SD (range)	4.65 ± 4.43 (1.0–23.75)
Donor type, living/deceased donor		20 (22%)/69 (78%)
Maintenance Immunosuppressive Therapy		
Steroid	*n* (%)	84 (94%)
Tacrolimus	*n* (%)	75 (84%)
Cyclosporine	*n* (%)	13 (15%)
Mycophenolate Mofetil	*n* (%)	77 (87%)
**PTDM Characteristics and Management**		
Duration PTDM, years	Median [IQR]	8.17 [4.42–14.25]
	Mean ± SD (range)	9.29 ± 6.64 (0.25–27)
Antidiabetic therapies pre-SGLT2-i/GLP-1 RA initiation		
Biguanides (Metformin)	*n* (%)	29/89 (33%)
Sulphonylureas	*n* (%)	10/89 (11%)
DPP4-i	*n* (%)	35/89 (39%)
SGLT2-i **	*n* (%)	----
GLP-1 RA **	*n* (%)	----
Insulin	*n* (%)	43/89 (48%)

Abbreviations: BMI: Body Mass Index; DM: Diabetes Mellitus; DPP4-i: Dipeptidyl Peptidase-4 Inhibitors; ESKD: End-Stage Kidney Disease; GLP-1 RA: Glucagon-Like Peptide-1 Receptor Agonists; KT: Kidney Transplantation; PTDM: Post-Transplant Diabetes Mellitus; KRT: Kidney Replacement Therapy; SGLT2-i: Sodium-Glucose Cotransporter-2 Inhibitors. Note: * The dialysis modalities are not mutually exclusive. ** SGLT2-i and GLP-1 RA were excluded from baseline analysis, as the study focuses on their introduction during treatment.

**Table 2:** Metabolic and Kidney Outcomes Before and After Initiating SGLT2i and/or GLP-1 RA Therapy (*n* = 89).

Variables	Measure	Baseline	End Follow-Up	*p*-Value *
HbA1c, %	mean ± SD	7.22 ± 1.18	7.01 ± 0.93	0.56
FPG, mg/dL	mean ± SD	112.62 ± 26.03	125.01 ± 30.60	**0.03**
TC, mg/dL	mean ± SD	175.53 ± 40.49	162.46 ± 41.14	**<0.01**
HDL-c, mg/dL	mean ± SD	48.69 ± 13.42	50.33 ± 12.84	0.07
LDL-c, mg/dL	mean ± SD	87.23 ± 37.22	80.92 ± 35.00	**0.02**
TG, mg/dL	mean ± SD	187.67 ± 104.03	179.72 ± 153.96	0.45
Weight, Kg	mean ± SD	73.95 ± 14.89	70.48 ± 13.67	**0.03**
BMI, Kg/m^2^	mean ± SD	27.27 ± 4.86	25.95 ± 4.15	**<0.001**
SBP, mmHg	mean ± SD	143.21 ± 17.31	137.18 ± 13.40	**<0.01**
DBP, mmHg	mean ± SD	76.27 ± 16.16	74.21 ± 10.41	0.08
Creatinine, mg/dL	mean ± SD	1.59 ± 0.58	1.57 ± 0.60	0.68
Urea, mg/dL	mean ± SD	74.94 ± 29.03	71.19 ± 29.34	0.14
Uric acid, mg/dL	mean ± SD	6.52 ± 1.42	6.18 ± 1.47	**0.03**
eGFR, mL/min/1.73 m^2^	mean ± SD	52.74 ± 20.61	54.81 ± 21.37	0.11

* Statistically significant *p*-values are highlighted in bold. Abbreviations: eGFR: Estimated Glomerular Filtration Rate; FPG: Fasting Plasma Glucose; HbA1c: Glycated Hemoglobin; TC: Total Cholesterol; HDL-c: High-Density Lipoprotein Cholesterol; LDL-c: Low-Density Lipoprotein Cholesterol; TG: Triglycerides; BMI: Body Mass Index; SBP: Systolic Blood Pressure; DBP: Diastolic Blood Pressure.

## Data Availability

The data presented in this study are available on request from the corresponding author. The data are not publicly available due to privacy and ethical restrictions.

## References

[1] Kanbay M., Siriopol D., Guldan M., Ozbek L., Topcu A.U., Siriopol I., Tuttle K. (2025). Prognostic impact of post-transplant diabetes mellitus in kidney allograft recipients: A meta-analysis. Nephrol. Dial. Transplant..

[2] Oliveras L., Coloma A., Lloberas N., Lino L., Favà A., Manonelles A., Codina S., Couceiro C., Melilli E., Sharif A. (2024). Immunosuppressive drug combinations after kidney transplantation and post-transplant diabetes: A systematic review and meta-analysis. Transplant. Rev..

[3] Montero N., Oliveras L., Soler M.J., Cruzado J.M. (2021). Management of post-transplant diabetes mellitus: An opportunity for novel therapeutics. Clin. Kidney J..

[4] Haidinger M., Antlanger M., Kopecky C., Kovarik J.J., Säemann M.D., Werzowa J. (2015). Post-transplantation diabetes mellitus: Evaluation of treatment strategies. Clin. Transplant..

[5] Munoz Pena J.M., Cusi K. (2023). Posttransplant Diabetes Mellitus: Recent Developments in Pharmacological Management of Hyperglycemia. J. Clin. Endocrinol. Metab..

[6] Zinman B., Wanner C., Lachin J.M., Fitchett D., Bluhmki E., Hantel S., Mattheus M., Devins T., Johansen O.E., Woerle H.J. (2015). Empagliflozin, Cardiovascular Outcomes, and Mortality in Type 2 Diabetes. N. Engl. J. Med..

[7] Herrington W.G., Staplin N., Wanner C., Green J.B., Hauske S.J., Emberson J.R., Preiss D., Judge P., Mayne K.J., The EMPA-KIDNEY Collaborative Group (2023). Empagliflozin in Patients with Chronic Kidney Disease. N. Engl. J. Med..

[8] Wiviott S.D., Raz I., Bonaca M.P., Mosenzon O., Kato E.T., Cahn A., Silverman M.G., Zelniker T.A., Kuder J.F., Murphy S.A. (2019). Dapagliflozin and Cardiovascular Outcomes in Type 2 Diabetes. N. Engl. J. Med..

[9] Heerspink H.J.L., Stefánsson B.V., Correa-Rotter R., Chertow G.M., Greene T., Hou F.F., Mann J.F.E., McMurray J.J.V., Lindberg M., Rossing P. (2020). Dapagliflozin in Patients with Chronic Kidney Disease. N. Engl. J. Med..

[10] Neal B., Perkovic V., Matthews D.R. (2017). Canagliflozin and Cardiovascular and Renal Events in Type 2 Diabetes. N. Engl. J. Med..

[11] Marso S.P., Daniels G.H., Brown-Frandsen K., Kristensen P., Mann J.F., Nauck M.A., Nissen S.E., Pocock S., Poulter N.R., Ravn L.S. (2016). Liraglutide and Cardiovascular Outcomes in Type 2 Diabetes. N. Engl. J. Med..

[12] Marso S.P., Baeres F.M.M., Bain S.C., Goldman B., Husain M., Nauck M.A., Poulter N.R., Pratley R.E., Thomsen A.B., Buse J.B. (2020). Effects of Liraglutide on Cardiovascular Outcomes in Patients with Diabetes with or Without Heart Failure. J. Am. Coll. Cardiol..

[13] Marso S.P., Bain S.C., Consoli A., Eliaschewitz F.G., Jódar E., Leiter L.A., Lingvay I., Rosenstock J., Seufert J., Warren M.L. (2016). Semaglutide and Cardiovascular Outcomes in Patients with Type 2 Diabetes. N. Engl. J. Med..

[14] Husain M., Birkenfeld A.L., Donsmark M., Dungan K., Eliaschewitz F.G., Franco D.R., Jeppesen O.K., Lingvay I., Mosenzon O., Pedersen S.D. (2019). Oral Semaglutide and Cardiovascular Outcomes in Patients with Type 2 Diabetes. N. Engl. J. Med..

[15] Gerstein H.C., Colhoun H.M., Dagenais G.R., Diaz R., Lakshmanan M., Pais P., Probstfield J., Riesmeyer J.S., Riddle M.C., Rydén L. (2019). Dulaglutide and cardiovascular outcomes in type 2 diabetes (REWIND): A double-blind, randomised placebo-controlled trial. Lancet.

[16] American Diabetes Association Professional Practice Committee (2022). 2. Classification and Diagnosis of Diabetes: Standards of Medical Care in Diabetes-2022. Diabetes Care.

[17] Sharif A., Chakkera H., de Vries A.P.J., Eller K., Guthoff M., Haller M.C., Hornum M., Nordheim E., Kautzky-Willer A., Krebs M. (2024). International consensus on post-transplantation diabetes mellitus. Nephrol. Dial. Transplant..

[18] (2024). Kidney Disease: Improving Global Outcomes (KDIGO) CKD Work Group. KDIGO 2024 Clinical Practice Guideline for the Evaluation and Management of Chronic Kidney Disease. Kidney Int..

[19] Delgado C., Baweja M., Crews D.C., Eneanya N.D., Gadegbeku C.A., Inker L.A., Mendu M.L., Miller W.G., Moxey-Mims M.M., Roberts G.V. (2022). A Unifying Approach for GFR Estimation: Recommendations of the NKF-ASN Task Force on Reassessing the Inclusion of Race in Diagnosing Kidney Disease. Am. J. Kidney Dis..

[20] Kukla A., Hill J., Merzkani M., Bentall A., Lorenz E.C., Park W.D., D’Costa M., Kudva Y.C., Stegall M.D., Shah P. (2020). The Use of GLP1R Agonists for the Treatment of Type 2 Diabetes in Kidney Transplant Recipients. Transplant. Direct.

[21] Halden T.A.S., Kvitne K.E., Midtvedt K., Rajakumar L., Robertsen I., Brox J., Bollerslev J., Hartmann A., Åsberg A., Jenssen T. (2019). Efficacy and Safety of Empagliflozin in Renal Transplant Recipients With Posttransplant Diabetes Mellitus. Diabetes Care.

[22] Sánchez Fructuoso A.I., Bedia Raba A., Banegas Deras E., Vigara Sánchez L.A., Valero San Cecilio R., Franco Esteve A., Cruzado Vega L., Gavela Martínez E., González Garcia M.E., Saurdy Coronado P. (2023). Sodium-glucose cotransporter-2 inhibitor therapy in kidney transplant patients with type 2 or post-transplant diabetes: An observational multicentre study. Clin. Kidney J..

[23] Schwaiger E., Burghart L., Signorini L., Ristl R., Kopecky C., Tura A., Pacini G., Wrba T., Antlanger M., Schmaldienst S. (2019). Empagliflozin in posttransplantation diabetes mellitus: A prospective, interventional pilot study on glucose metabolism, fluid volume, and patient safety. Am. J. Transplant..

[24] Kim H.S., Lee J., Jung C.H., Park J.Y., Lee W.J. (2021). Dulaglutide as an Effective Replacement for Prandial Insulin in Kidney Transplant Recipients with Type 2 Diabetes Mellitus: A Retrospective Review. Diabetes Metab. J..

[25] Vigara L.A., Villanego F., Orellana C., Naranjo J., Torrado J., Garcia T., Mazuecos A. (2022). Effectiveness and safety of glucagon-like peptide-1 receptor agonist in a cohort of kidney transplant recipients. Clin. Transplant..

[26] Sato T., Azuma Y., Ozone C., Okazaki M., Takeda A., Okada M., Futamura K., Hiramitsu T., Goto N., Narumi S. (2023). Possible Advantage of Glucagon-Like Peptide 1 Receptor Agonists for Kidney Transplant Recipients With Type 2 Diabetes. J. Clin. Endocrinol. Metab..

[27] Mallik R., Ali O., Casabar M., Mukuba D., Byrne C., McCafferty K., Yaqoob M.M., Chowdhury T.A. (2023). Glucagon-like peptide-1 receptor analogues in renal transplant recipients with diabetes: Medium term follow of patients from a single UK centre. Diabet. Med..

[28] Lemke A., Brokmeier H.M., Leung S.B., Mara K.C., Mour G.K., Wadei H.M., Hill J.M., Stegall M., Kudva Y.C., Shah P. (2022). Sodium-glucose cotransporter 2 inhibitors for treatment of diabetes mellitus after kidney transplantation. Clin. Transplant..

[29] Song C.C., Brown A., Winstead R., Yakubu I., Demehin M., Kumar D., Gupta G. (2020). Early initiation of sodium-glucose linked transporter inhibitors (SGLT-2i) and associated metabolic and electrolyte outcomes in diabetic kidney transplant recipients. Endocrinol. Diabetes Metab..

[30] Pinelli N.R., Patel A., Salinitri F.D. (2013). Coadministration of liraglutide with tacrolimus in kidney transplant recipients: A case series. Diabetes Care.

[31] Liou J.H., Liu Y.M., Chen C.H. (2018). Management of Diabetes Mellitus With Glucagonlike Peptide-1 Agonist Liraglutide in Renal Transplant Recipients: A Retrospective Study. Transplant. Proc..

